# Infrared Spectroscopy: A New Frontier in Hematological Disease Diagnosis

**DOI:** 10.3390/ijms242317007

**Published:** 2023-11-30

**Authors:** Charlotte Delrue, Reinhart Speeckaert, Matthijs Oyaert, Tessa Kerre, Sylvie Rottey, Renaat Coopman, Wouter Huvenne, Sander De Bruyne, Marijn M. Speeckaert

**Affiliations:** 1 Department of Nephrology, Ghent University Hospital, 9000 Ghent, Belgium; charlotte.delrue@ugent.be; 2 Department of Dermatology, Ghent University Hospital, 9000 Ghent, Belgium; reinhart.speeckaert@ugent.be; 3 Department of Clinical Biology, Ghent University Hospital, 9000 Ghent, Belgium; matthijs.oyaert@uzgent.be (M.O.); sander.debruyne@uzgent.be (S.D.B.); 4 Department of Hematology, Ghent University Hospital, 9000 Ghent, Belgium; tessa.kerre@ugent.be; 5 Department of Medical Oncology, Ghent University Hospital, 9000 Ghent, Belgium; sylvie.rottey@ugent.be; 6 Department of Oral, Maxillofacial and Plastic Surgery, Ghent University Hospital, 9000 Ghent, Belgium; renaat.coopman@uzgent.be; 7 Department of Head and Neck Surgery, Ghent University Hospital, 9000 Ghent, Belgium; wouter.huvenne@ugent.be; 8 Research Foundation-Flanders (FWO), 1000 Brussels, Belgium

**Keywords:** infrared spectroscopy, hematological diseases, fingerprint

## Abstract

Hematological diseases, due to their complex nature and diverse manifestations, pose significant diagnostic challenges in healthcare. The pressing need for early and accurate diagnosis has driven the exploration of novel diagnostic techniques. Infrared (IR) spectroscopy, renowned for its noninvasive, rapid, and cost-effective characteristics, has emerged as a promising adjunct in hematological diagnostics. This review delves into the transformative role of IR spectroscopy and highlights its applications in detecting and diagnosing various blood-related ailments. We discuss groundbreaking research findings and real-world applications while providing a balanced view of the potential and limitations of the technique. By integrating advanced technology with clinical needs, we offer insights into how IR spectroscopy may herald a new era of hematological disease diagnosis.

## 1. Introduction

Hematological diseases represent various conditions that affect the blood and blood-forming organs. These disorders affect millions worldwide, from conditions such as anemia, which leads to chronic fatigue due to a reduced red blood cell count, to malignancies such as leukemia and lymphoma characterized by abnormal cell growth. Traditional diagnostic methods, although effective, have limitations. Although informative, bone marrow biopsies are invasive and often painful. Blood tests can be laborious and sometimes require multiple iterations for conclusive diagnosis. These methods’ economic and temporal costs pose significant challenges, particularly in areas with constrained healthcare resources.

Infrared (IR) spectroscopy is a diagnostic technique that provides a “special fingerprint” by measuring the molecular vibrational energy. This method promises to detect molecular abnormalities in blood without invasive procedures. Coupled with technological advancements, IR spectroscopy can provide swift results. However, its adoption in hematological diagnostics is not without challenges, such as the need for specialized expertise in spectral data interpretation and potential sensitivity issues.

Despite these obstacles, the myriad advantages of IR spectroscopy (its noninvasiveness, speed, and cost-effectiveness) position it at the forefront of contemporary hematological research. This review discusses the role of IR spectroscopy in hematological diagnostics and explores its current capabilities, challenges, and potential to transform the detection and management of blood diseases.

## 2. Fundamentals of Infrared Spectroscopy

IR spectroscopy is based on the interaction between IR radiation and molecular vibrations in a sample, producing a unique “signature”. The intensity of IR spectra provides quantitative data, while absorption locations (in terms of wavelength or wavenumber) reveal qualitative details about chemical bonds and their molecular environments. In complex biological substances, major IR absorption bands often arise from bonds in proteins, lipids, and nucleic acids, such as N-H, C=O, C-H, and P=O [1].

IR spectroscopy encompasses various techniques with distinct wavelength ranges and advantages. Mid-infrared (MIR) spectroscopy, spanning from 4000 to 400 cm^−1^, is renowned for its sensitivity to fundamental molecular vibrations, making it particularly useful for detailed molecular characterization. Fourier transform infrared spectroscopy (FTIR), a type of MIR spectroscopy, offers high-resolution spectra and is frequently used for qualitative analysis of blood samples. In the higher wavelength range, near-infrared (NIR) spectroscopy, which covers from 14,000 to 4000 cm^−1^, stands out for quantitative analyses because of its ability to penetrate deeper into the samples. This depth facilitates more comprehensive sample insights, making NIR a preferred choice for certain applications. Attenuated total reflection (ATR) specializes in probing the surface properties of samples, revealing information about surface-bound molecules and potential contaminants or modifications. Regardless of the specific technique, IR spectroscopy has numerous advantages, such as rapid analysis, noninvasiveness, minimal sample preparation, and versatility in handling a wide array of hematological samples.

A century has passed since William Herschel’s initial discoveries, but only recently has IR spectroscopy been refined enough for cellular and molecular examinations crucial to blood functionality. For instance, using IR to screen and diagnose β-thalassemia carriers offers advantages such as reagent-free methods, minute sample requirements, automation potential, short analysis time, and ease of learning [2]. However, IR spectroscopy has its challenges, including sample variability, instrument calibration, and the need for standardized protocols. Its sensitivity can also fluctuate based on the specific hematological conditions under study.

## 3. Hematological Diseases

### 3.1. Anemia

**Figure 1:**
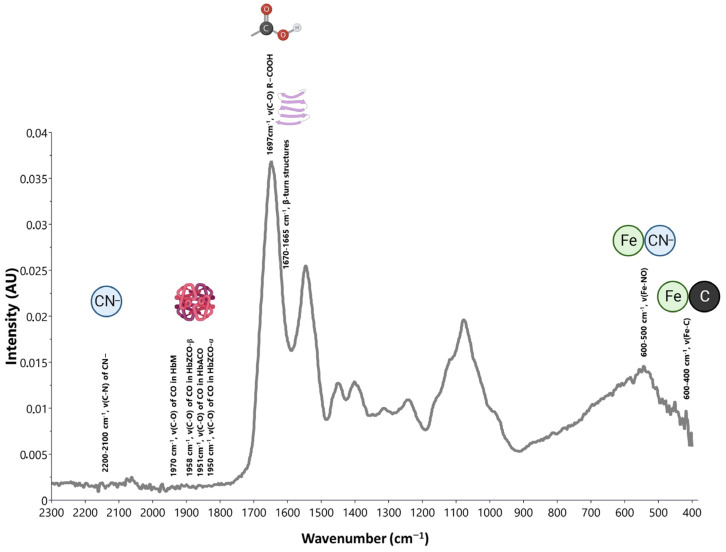
Infrared spectra of blood samples illustrating the contributing wavenumbers for differentiating anemia patients from healthy controls. The spectra highlight distinct variations in hemoglobin structure, secondary protein structures and variations in the Fe-O stretching mode. Abbreviations: v: stretching; δ: bending; s: symmetric; and as: asymmetric.

Anemia, characterized by a deficiency in the number or quality of red blood cells, is a prevalent condition with diverse underlying causes, from nutrient deficiencies to genetic mutations. The molecular complexities associated with anemia have made it a focal point of extensive research, especially in the field of advanced diagnostic techniques. In this context, IR spectroscopy stands out as a promising tool, offering noninvasive, rapid, and detailed analysis of hemoglobin (Hb) structures and abnormalities. Through IR spectroscopy, researchers can detect subtle molecular changes, providing insights into the nature and cause of specific anemic conditions (Figure 1). The subsequent sections delve deeper into the specificities of IR spectroscopic examinations of Hb, underscoring its potential to enhance our understanding and management of anemia.

IR spectroscopic examinations of Hb have concentrated on the pronounced carbon monoxide (CO) stretching IR band when CO is bound to Hb (HbCO) [3], faint yet distinct SH stretching bands of cysteine residues [4], and vibrations from the side chain and backbone of the protein [5,6]. This technique has been proven effective in distinguishing CO binding to different subunits in human Hb mutants. Notably, significant alterations in the heme environment lead to unique spectral bands for CO bound to α- and β-subunits [3]. The task of attributing distinct SH stretching bands in the IR spectrum of human Hb to specific cysteine residues was masterfully performed by contrasting it with the spectrum of horse Hb, which lacks cysteine β-112, and with bovine Hb, known for only having β-93 cysteine [4]. The SH stretch vibrations of cysteine residues in HbA react with structural alterations caused by the attachment of ligands such as O_2_, CO, and NO to the heme iron [2,7]. When comparing the IR difference spectrum of HbA and Hb Kempsey (a mutation where Asp β-99 is substituted by Asn), a negative band at 1697 cm^−1^ was observed. This band, associated with the CO stretch of carboxylic acid, was attributed to the side chain of Asp β-99 owing to its recognized mutation [5]. Wallace et al. showed that the vCO of carbon monoxide attached to the α- and β-chains in Hb Zurich (HbZ, β-63 His-Arg) transitioned to 1950 and 1958 cm^−1^, respectively, in contrast with the standard 1951 cm^−1^ (HbACO) [6]. Modification of the distal histidine (β-63) in HbA to arginine in HbZ increases its vulnerability to autooxidation when exposed to specific oxidizing agents. This insight linked the structure with pathological characteristics of HbZ and bolstered the argument that IR spectroscopy can illuminate the causes of Hb disorders stemming from irregular Hb structures [2].

The IR spectroscopy of Hb, with a specific focus on the heme iron complex, has significant diagnostic potential in the context of hematological disorders. This technique revealed distinct IR absorption features attributable to the vibrational modes of the heme group and its iron-bound ligands. These spectral characteristics are pivotal in indicating the state of heme iron, including its oxidation state and coordination environment, which undergo alterations in various blood pathologies. In a study of two M-type Hbs, Boston (His alpha 58-->Tyr) and Saskatoon (His beta 63-->Tyr), the abnormal subunits of both Hbs displayed IR spectral bands near 1970 cm^−1^, in contrast to the normal subunits which showed bands near 1951 cm^−1^. This disparity in wavenumbers was significant for identifying the abnormal subunits. Additionally, the study measured the extent of abnormal subunit oxidation by observing the intensity increase in the 1970 cm^−1^ band following erythrocyte reduction with dithionite [8]. The IR spectra of Hb are influenced by the binding of ligands, such as O_2_ and CO, to heme iron (II) in its deoxy form, as well as by its oxidation to form aquoiron (III) complexes. Spectral analyses across human, bovine, and equine Hbs, particularly in phosphate buffer solutions, have shown consistent spectral shifts in the amide I region, specifically around 1665–1670 cm^−1^. These shifts are associated with β-turn structures in the proteins. Importantly, these analyses revealed that significant secondary structural changes do not occur in the amide I region, underscoring the stability of these structures across different species and conditions. The stability and consistency are vital for the application of IR spectroscopy in clinical diagnostics and the understanding of the behavior of Hb in various pathological states [9].

The binding of nitric oxide (NO) to Hb induces detectable structural changes, primarily through shifts in molecular vibrations. A crucial absorption band is the Fe-NO stretching mode, indicative of NO binding to iron in the heme group, which appears in the 500–600 cm^−1^ region [10,11,12]. Additionally, the N-O stretching vibration of NO, typically falling within 1500–1900 cm^−1^, can shift slightly depending on the bonding environment of Hb. The heme group itself, encompassing various vibrational modes, including C-H, C-N, and C-C stretching and bending modes, also undergoes changes. These modes generally appear in the fingerprint region (below 1500 cm^−1^) and the functional group region (1500–4000 cm^−1^) [13,14]. The binding of cyanide to Hb results in distinct changes in the IR spectrum, with a focus on the C-N stretching vibration of the cyanide ion, typically appearing in the 2100–2200 cm^−1^ region. The exact position of this band can vary depending on the bonding environment of Hb [15,16,17]. Additionally, the iron–cyanide (Fe-C) stretching vibration is a significant marker, usually found in the lower-frequency range of 400–600 cm^−1^, which differs from Fe-O stretching in oxyhemoglobin. In addition, changes were observed in the vibrational modes of the heme group, including various C-H, C-N, and C-C stretching and bending modes. These alterations are typically observed in the fingerprint (below 1500 cm^−1^) and functional group region (1500–4000 cm^−1^) [14,18,19]. Exact wavenumbers can vary based on the state of Hb, environmental conditions, and experimental settings, necessitating specific studies for precise analysis and interpretation in different contexts.

Red cell transfusions aim to enhance the oxygen supply to body tissues. While research has been conducted on Hb concentration levels as indicators of transfusion, the optimal Hb level for clinical advantage remains ambiguous. In a systematic review, noninvasive NIR spectroscopy was investigated as a potentially superior indicator for transfusion to directly gauge tissue oxygenation. The significance of tissue oxygenation in predicting transfusion was established in only four studies, all of which focused on muscle oxygen saturation in the context of adult trauma. The overall detection rate was modest, 34% (27–42%), and although the specificity was relatively higher at 78% (74–82%), the varied and retrospective methodologies introduced significant ambiguity to these results. Four forward-looking randomized studies encompassing 540 patients in fields such as cardiac and neurological surgery as well as neonatal care juxtaposed NIR-spectroscopy-guided transfusion decisions against conventional methods. The results indicated a decline in the volume of red cells per individual (odds ratio (OR): 0.44 (0.09–0.79)), but not in the number of individuals receiving transfusion (OR: 0.71 (0.46–1.10)), with no notable shift in health outcomes. While assessing tissue oxygen saturation shows promise in informing transfusion decisions, the current data pool is insufficient to advocate its broad-based adoption in clinical settings. A call for measurement standardization and further investigation into the thresholds where tissue oxygenation might result in negative health outcomes would pave the way for upcoming clinical studies [20].

### 3.2. Leukemia

In the 1950s, Polli et al. employed IR spectroscopy to study DNA extracted from regular and leukemic human leukocytes. However, no distinct spectroscopic variations were observed between the two types of nucleic acids [21]. Subsequent to advancements in IR spectroscopy hardware and techniques, Benedetti et al. conducted a series of refined experiments to delve deeper into the spectral variations between normal and leukemic cells [22]. Two DNA marker bands were identified in the IR spectrum, specifically at 966 cm^−1^ and 530 cm^−1^, which are characteristic of lymphoid leukemia. Moreover, the proportion between two encompassed spectral zones (the symmetric PO^2−^ stretching absorption band at 1080 cm^−1^ and the amide II absorption band at 1540 cm^−1^) served to differentiate normal lymphocytes from leukemic cells [23]. Instead of depending on singular marker bands in the IR spectra of leukemic cells, pattern recognition algorithms have been developed, which harness all fingerprint-like features present in the IR spectrum [24].

In a study of 30 leukemia patients (22 acute myeloid leukemia (AML), 4 chronic myeloid leukemia (CML), and 4 acute lymphoblastic leukemia (ALL)) and 19 healthy controls, the peak height ratio of 2959 cm^−1^/2931 cm^−1^, which represents CH_3_/CH_2_, demonstrated the most substantial and statistically significant difference. The 2959 cm^−1^ band corresponds to the asymmetric C-H stretching in methyl groups, while the 2931 cm^−1^ band arises from asymmetric C-H stretching in methylene groups. FTIR spectroscopy exhibited a sensitivity and specificity of 83.3% and 79%, respectively. This suggests that it could potentially serve as a valuable factor for distinguishing between the serum of patients with leukemia and that of healthy individuals. Moreover, curve fitting analysis revealed that the RNA (1115 cm^−1^)/DNA (1028 cm^−1^) ratios (A1115/A1028) were notably lower in the serum of patients with leukemia than in the serum of healthy individuals [25].

#### 3.2.1. Acute Lymphoblastic Leukemia

ALL is the most common cancer in children, accounting for approximately 25% of all cancer diagnoses in children up to 15 years of age. ALL originates from abnormal transformation and growth of lymphoid precursor cells in the bone marrow. In children, it typically stems from B-cell precursors, representing approximately 80% of all cases (BCP-ALL) [26].

**Figure 2:**
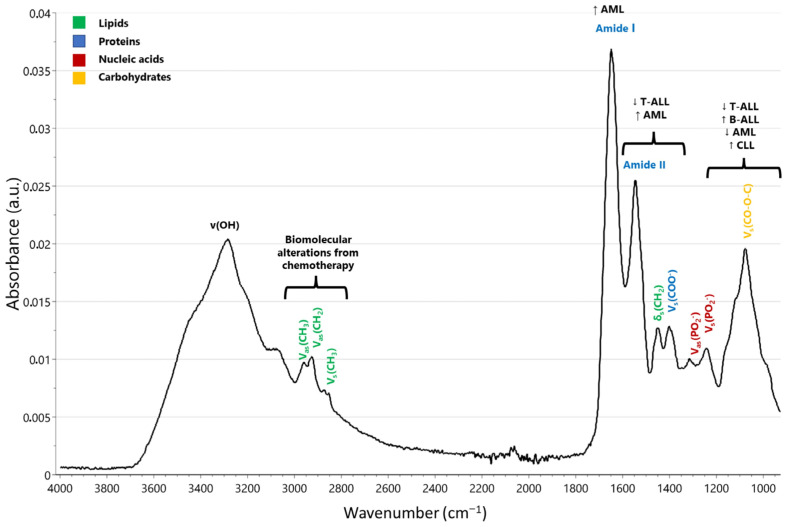
Infrared spectra illustrating differences among various leukemic patients. The spectra highlight distinct variations in protein signatures, evident from the amide I and II bands, as well as nucleic acid content. Also, biomolecular alterations induced by chemotherapy in the lipid region are illustrated. Abbreviations: AML: acute myeloid leukemia; T-ALL: T-cell acute lymphoblastic leukemia; B-ALL: B-cell acute lymphoblastic leukemia; CLL: chronic lymphocytic leukemia. Abbreviations: v: stretching; δ: bending; s: symmetric; and as: asymmetric.

In the study of Ramesh et al. (Table 1), Fourier transform infrared microspectroscopy (micro-FTIR) analysis of isolated peripheral lymphocytes distinguished T-cell ALL patients from the control group through absorbance alterations, characterized by a significant reduction in protein content among patients. The most notable spectral variations were observed within the 1000–1200 cm^−1^ region, aligned with the presence of nucleic acids in the cells. The higher-wavenumber region spanning from 2800–3000 cm^−1^ offered valuable insights into biomolecular alterations resulting from chemotherapy (Figure 2). In B-cell ALL cases, the reduction in integrated absorbance during chemotherapy may be attributed to a significant decrease in nucleic acids or phospholipids, as there were no observable changes in the amide II band, indicating no major alterations in protein content within the cells. In T-cell ALL cases, the decrease in integrated absorbance in samples prior to treatment could be linked to a decline in the protein or phospholipid content. This interpretation is supported by the decrease in absorbance within the amide II band in pre-treatment samples compared to the controls, while the protein content remained relatively stable throughout the course of chemotherapy treatment. There was an immediate decrease in both DNA and RNA levels following the initiation of methotrexate chemotherapy. This reduction in DNA content was corroborated by a concurrent decrease in the total phosphate content. Furthermore, the spectra displayed a notable reduction in absorption at 965 and 1245 cm^−1^, which are associated with phosphodiester bonds present in nucleic acids [27].

In a case series study, the spectra of peripheral blood mononuclear cells (PBMCs) from leukemia patients were compared with those of patients with infection (exhibiting “flu-like” symptoms similar to leukemia) and healthy individuals. Additionally, micro-FTIR spectroscopy was employed for the long-term monitoring of patients with leukemia. Spectral data derived from PBMCs were assessed in conjunction with routine tests for the presence of blasts in the bone marrow to assess the efficacy of chemotherapy and the compatibility of this novel technique with established methodologies. The most evident and pronounced distinctions among the spectra obtained from 15 leukemia patients, 19 patients with “infection” symptoms resembling leukemia, and 27 control individuals were linked to the bands related to lipids and proteins within the 3000–2800 cm^−1^ region [28]. A plausible rationale for the unusually reduced lipid absorption in the PBMCs of leukemia patients could be attributed to heightened fluidity and altered lipid composition within the plasma membrane of blast cells compared to healthy cells [29,30,31]. Additionally, systemic factors such as serum lipid content, which is known to influence membrane fluidity and the overall composition of PBMCs, may also play a role in these observations [32,33]. It is widely recognized that lipid composition within the plasma membrane has a substantial influence on drug resistance [34]. Furthermore, several supplementary biochemical markers, including DNA, have been identified and statistically validated as effective diagnostic variables for the identification of childhood leukemia [28]. The absorption of DNA showed a decline that corresponded to the duration of treatment. This is thought to be linked to the rapid reduction in blast cells during the early days of treatment, which contributes to the initially elevated IR absorption of DNA. Additionally, this decline may be associated with the presence of apoptotic blasts/PBMCs, where chromatin condensation during apoptosis increases the opacity of DNA to IR light. This heightened opacity results in a decrease in the actual signal within the absorption band attributed to DNA [35].

In addition to the examination of isolated peripheral lymphocytes, FTIR spectroscopy was employed to distinguish between newly diagnosed ALL cases and disease-free bone marrow samples. The analysis revealed distinct alterations in the characteristic bands in both healthy and diseased samples, originating from cellular proteins, lipids, and DNA. Notably, specific changes affecting the secondary structure of proteins were evident in the FTIR spectra and confirmed through second-derivative analysis. In the control samples, the predominant protein structure consisted primarily of α-helices, whereas in ALL samples, a relatively higher proportion of antiparallel β-sheet protein constituents (1688 cm^−1^) was observed, likely due to the presence of leukemia. Various absorbance ratios for specific bands were calculated and plotted against the patient samples. These ratios exhibited significant variations, reflecting changes in the biomolecular structure between normal and leukemic samples. Frequency shifts were observed in several regions: amides A and B, amide I, and specific bands at 2927 cm^−1^ and 2869 cm^−1^. Moreover, the ratios of lipid/protein, amide II to amide I, and the intensity of amide I to the intensity at 2958 cm^−1^ all showed potential variations. These shifts and ratio variations could serve as valuable biomarkers for distinguishing between leukemia-free and leukemia samples [36].

In a small cohort study comprising 10 children diagnosed with BCP-ALL and 10 healthy control subjects, researchers used FTIR spectroscopy to analyze serum samples. Their analyses revealed several noteworthy findings. Firstly, there was a significant difference in the peak area ratio at 2965/1645 cm^−1^ between the BCP-ALL patients and the healthy controls, with a *p*-value of 0.002. This suggests that there are distinct molecular or structural differences between the sera of patients with BCP-ALL and healthy individuals. Additionally, patients with BCP-ALL exhibited a lower average percentage of both β-sheet and β-turn protein structures in their sera. This alteration in protein structure may provide insights into the underlying changes in serum composition associated with BCP-ALL [37]. This is in contrast with a previous study on free bone marrow samples, which indicated a relatively high presence of antiparallel β-sheet proteins in patients with ALL. It is important to note that this variation in results could stem from the distinct nature of the tissue under analysis, specifically comparing serum to bone marrow cells [36]. Variations in the composition of sera from individuals with leukemia contribute to the accelerated growth of leukemic cells in both the bloodstream and bone marrow. Compared with control subjects, patients with acute leukemia exhibited distinct metabolic differences in their serum. These differences encompass irregularities in metabolic pathways, such as glycolysis, tricarboxylic acid (TCA) cycle, alterations in lipoproteins, choline metabolism, and fatty acid metabolism [38,39]. Furthermore, an AdaBoost-based predictive model achieved an 85% accuracy rate in classifying individuals as either healthy or afflicted with BCP-ALL. This suggests that FTIR spectroscopy data could potentially be used for diagnostic purposes to distinguish between the two groups. Lastly, there was an interesting correlation observed between the phase shift of the first derivative in the spectral range of 1050–1042 cm^−1^ and white blood cell and blast cell counts in BCP-ALL patients, which was not evident in the samples from healthy controls. This correlation may offer insights into disease progression and severity. Although larger studies are needed to confirm these findings, the results are promising. FTIR spectroscopy may hold potential as a noninvasive and early screening tool for BCP-ALL. Detecting BCP-ALL at an early stage could enable timely intervention and treatment, potentially improving outcomes in affected children [37].

**Table 1:** Overview of studies on infrared spectroscopy in childhood acute lymphoblastic leukemia.

Study Population	Sample and Main Technique	Key Spectral Findings	Ref
Childhood ALL (T-cell and B-cell precursors)	Peripheral lymphocytes Micro-FTIR spectroscopy	- Micro-FTIR analysis distinguished T-cell ALL patients from controls through absorbance alterations in the range of 1300–1600 cm^−1^. - Notable spectral variations were observed in the 1000–1200 cm^−1^ region (related to nucleic acids). - Biomolecular alterations resulting from chemotherapy were observed in the higher-wavenumber region spanning from 2800–3000 cm^−1^. - Notable reduction in absorption at 965 and 1245 cm^−1^ associated with phosphodiester bonds in nucleic acids. - Reduced integrated absorbance during chemotherapy in B-cell ALL cases may be linked to decreased nucleic acids or phospholipids, with no major alterations in protein content. - Reduced integrated absorbance in T-cell ALL cases prior to treatment could be linked to decreased protein or phospholipid content. - Immediate decrease in DNA and RNA levels following chemotherapy initiation, with corroborated reduction in total phosphate content.	[27]
Leukemia patients, patients with “infection” symptoms resembling leukemia, and healthy individuals	PBMCs Micro-FTIR spectroscopy	- Distinct spectral differences related to lipids and proteins in the 3000–2800 cm^−1^ region among leukemia patients, “infection” patients, and healthy individuals. - Reduced lipid absorption in leukemia patients possibly attributed to altered lipid composition in plasma membrane of blast cells. - Biochemical markers, including DNA, identified and statistically validated for childhood leukemia diagnosis. - Decrease in DNA absorption linked to rapid reduction in blast cells during early chemotherapy and chromatin condensation in apoptotic blasts/PBMCs.	[28]
Childhood ALL vs. healthy controls	Bone marrow FTIR spectroscopy	- Evidence of distinct alterations in characteristic bands related to cellular proteins, lipids, and DNA in both healthy and diseased samples. - Structural changes in protein secondary structure with a higher proportion of antiparallel β-sheet protein constituents in ALL samples. - Various absorbance ratios indicating changes in biomolecular structure as potential biomarkers. - Frequency shifts observed at specific wavenumbers in the FTIR spectra.	[36]
BCP-ALL patients vs. healthy controls	Serum FTIR spectroscopy	- Significant difference in the peak area ratio at 2965/1645 cm^−1^ between BCP-ALL patients and healthy controls, indicating distinct structural differences in sera. - Lower average percentage of both β-sheet and β-turn protein structures in sera of BCP-ALL patients. - Development of a predictive model achieving an 85% accuracy rate in classifying individuals as healthy or afflicted with BCP-ALL. - Correlation observed between phase shift of the first derivative in the spectral range of 1050–1042 cm^−1^ and white blood cell and blast cell count in BCP-ALL patients, potentially providing insights into disease progression and severity.	[37]

Abbreviations: ALL, acute lymphoblastic leukemia; BCP, B-cell precursor; micro-FTIR spectroscopy, Fourier transform infrared microspectroscopy; PBMCs, peripheral blood mononuclear cells.

#### 3.2.2. Acute Myeloid Leukemia

Acute myeloid leukemia (AML) is a type of blood cancer characterized by excessive growth of myeloid precursor cells (elevated myeloblasts). This abnormal cell proliferation replaces healthy bone marrow with cancerous cells, resulting in compromised blood cell production and alterations in biochemical processes [40]. FTIR spectroscopy could be a valuable tool for the rapid and convenient identification of leukemia in peripheral blood.

Certain FTIR spectroscopic signatures of three leukemia cell lines (U937, HL-60, and THP-1) corresponding to the M2, M4, and M5 subtypes have been identified. These cell lines exhibited characteristics similar to those associated with leukemia. Specifically, there was a reduction in the presence of proteins in the α-helical structures (1657 cm^−1^ and 1650 cm^−1^), accompanied by an increase in proteins adopting β-sheets (1686 cm^−1^ and 1635 cm^−1^) and turn/loop structures (1673 cm^−1^ and 1663 cm^−1^) within the amide I region. Furthermore, an increase in the Z-backbone conformers of N-type sugars in nucleic acids (1416–1395 cm^−1^) and an overall increase in DNA concentration (1337 cm^−1^) were observed. Additionally, increases in the levels of glutamic acid (1700 cm^−1^) and tryptophan (1315 cm^−1^), along with a decrease in serine (1210 cm^−1^, 1194 cm^−1^, and 1011 cm^−1^) content across all three cell lines, were identified. These findings are consistent with established biochemical studies on AML. Further comparison of the spectroscopic signatures of peripheral blood with those of bone marrow using principal component analysis (PCA) revealed consistent leukemia-related components between peripheral blood and bone marrow in AML at spectral depths of 1604 cm^−1^ (related to the change in amino acid residues) and 1536 cm^−1^ (protein secondary structures of amide II). Based on the total count of all leukemia-related features at 1629 cm^−1^, 1610 cm^−1^, 1604 cm^−1^, 1536 cm^−1^, and 1528 cm^−1^ depths, along with the 1404 cm^−1^ peak in the peripheral blood and bone marrow, it was possible to perfectly categorize AML patients and healthy individuals [41].

Utilizing a multiplatform spectroscopic approach to investigate the impact of bezafibrate and medroxyprogesterone acetate (BaP) on HL60 and K562 cell lines provides a comprehensive view of how drug therapy affects the biochemical characteristics of these cells. This approach aids in gaining insight into the specific targets of BaP. Data obtained from synchrotron radiation FTIR (S-FTIR), ATR-FTIR, and Raman microspectroscopy strongly suggest that the biochemistry of lipids is a significant target of BaP. Furthermore, S-FTIR, which can be used to analyze hydrated cells, allowed the exploration of the interaction between the drug and DNA, revealing that DNA is unlikely to be a target of this therapy. Notably, an increase in methylene functionality after drug treatment indicated that AML cells exhibited increased unsaturation, resembling the typical biochemistry of noncancerous cells following BaP treatment. Raman microspectroscopy corroborated the FTIR findings and added an extra dimension by providing spatial information about lipid distribution. These spatial data suggest that the changes in saturation induced by BaP were consistent throughout a single cell [42].

Acute promyelocytic leukemia is set apart from other forms of leukemia owing to a balanced t(15;17) (q22;21) chromosome translocation, unique cellular morphology, and distinct clinical characteristics. All-trans-retinoic acid (ATRA) is a highly effective treatment for this condition, leading to the suppression of cell proliferation and inhibition of terminal granulocytic differentiation [43]. FTIR spectroscopy can be used to monitor the biochemical processes occurring during various stages of ATRA-induced NB4 cell differentiation. The significant and distinguishable spectral shifts in lipids (3200–2700 cm^−1^), nucleic acids, and carbohydrates (1300–900 cm^−1^) present opportunities to generate testable hypotheses. The peak centered at 968 cm^−1^ can be attributed to C-C/C-O stretching vibrations, primarily originating from the distinctive deoxyribose and phosphate components of the DNA backbone. Additionally, there were two other peaks centered at 1086 cm^−1^ and 1240 cm^−1^, arising from the symmetric and asymmetric stretching vibrations of PO^2-^ within the phosphodiester linkages found in the polynucleotide chains. Notably, the overall intensity in the region associated with nucleic acids/carbohydrates (940–1142 cm^−1^) displayed a significant increase compared to the amide bands (1480–1590 cm^−1^, amide II) following differentiation induction. The area ratio (A1085/A1545) exhibited a strong positive correlation with the differentiation index, calculated based on nitro blue tetrazolium (NBT) reduction and CD11b expression tests. Some of these spectroscopic parameters may hold promise as diagnostic indicators, aiding treatment decisions for patients with acute promyelocytic leukemia [44].

#### 3.2.3. Chronic Lymphocytic Leukemia

Chronic lymphocytic leukemia (CLL) is characterized by the buildup of nonproliferating, mature lymphocytes in the peripheral blood. As the disease advances, it can lead to lymphadenopathy, splenomegaly, and infiltration of leukemic cells into the bone marrow [45]. While every impacted cell deviates from its normal state, tumor cells exhibit a vast range of differences in shape, chromosomal makeup, and numerous other aspects. Given that IR spectra offer a unique signature for all biomolecules in a cell, IR spectroscopy has the capability to detect phenotypic changes in CLL cells beyond what can be visually observed on blood smear slides [46].

Thirty-eight patients with CLL underwent FTIR spectroscopy analysis (spectral range between 900 and 1300 cm^−1^), which showed higher DNA content and lower lipid content in CLL cells than in normal cells (Table 2). This spectral range captures the differences in the absorption of phosphate and sugar in DNA structures. In particular, band 1 was derived from the C-C/C-O stretching vibration associated with the distinctive deoxyribose and phosphate components of the DNA backbone. Meanwhile, band 2 (at 1087 cm^−1^) and band 3 (at 1240 cm^−1^) arise from the symmetric and asymmetrical PO^2−^ stretching vibrations of phosphate groups integral to the DNA structure. A statistical analysis employing hierarchical clustering effectively segregated normal cells from CLL cells and classified them into two distinct subgroups for normal cells. In contrast, CLL cells displayed greater heterogeneity, as they could be categorized into three distinct subgroups that were discernibly different from normal cell clusters. These distinctions were primarily attributed to variations in lipid and DNA content as well as the overall spectral characteristics of the cells [24]. In agreement with the findings reported by Benedetti et al. [22,23,47] concerning both cellular and isolated nuclei, this study corroborated that CLL cells exhibit higher phosphate content, which is primarily attributed to elevated DNA content. Discernible spectral alterations in the overall shape of these bands suggest potential structural or conformational changes within DNA. The increased DNA content observed in leukemic cells is likely associated with chromosomal abnormalities, affecting approximately 56–65% of patients with CLL. Specifically, trisomy 12 occurs in 18% of cases, while 14q+ and 13q+ abnormalities are identified in 13% and 10% of cases, respectively [48]. In another study, micro-FTIR spectroscopy was employed to analyze human plasma samples, including those from individuals afflicted with CLL. The findings revealed that spectral peaks at 1056 cm^−1^ (carbohydrates), 1270 cm^−1^ (amide III band), and 1592 cm^−1^ (δ(NH2): amino acids), consistently present in all samples from healthy individuals, exhibited significant reductions in all samples from the tested patients. Cluster analysis of the spectra acquired from these specific regions resulted in excellent classification accuracy, perfectly aligned with the clinical data, and effectively distinguished between healthy and patient samples [49].

By focusing on a distinct spectral region to distinguish cell changes in CLL (related to DNA base pairing), it is possible to track disease progression in patients over time and after medication. Ramesh et al. utilized IR spectroscopy to monitor the chemotherapy treatment in children with B- and T-cell leukemia [27]. They verified that the main spectral shifts in leukemic cells appeared in the range between 1000 and 1200 cm^−1^, relating to the cellular nucleic acids. Significantly, a consistent correlation was observed between the decrease in nucleic acid content, as identified by IR spectral analysis, and the decrease in blast percentage, a result of chemotherapy, in both B- and T-cell patients. The relative strength of the DNA band, characteristic of base pairing at 1713 cm^−1^, appears to signal the intensity of the disease [46]. IR spectroscopy can serve as an innovative method for forecasting ex vivo drug reactions, including drug sensitivity and resistance. The alterations in the spectrum revealed that the primary cellular modifications causing differences between drug-sensitive and drug-resistant cells were attributed to (a) lipids, (b) sugars/phosphates (DNA/RNA), and (c) proteins [50,51,52].

### 3.3. Lymphoma

Identifying and accurately classifying lymphoma ranks among the most difficult responsibilities in contemporary pathology. This challenge intensifies as tissue samples become smaller and the requisite range of analyses for subtyping expands. Although histological examination of tissue sections remains the primary method for lymphoma diagnosis, it is often complemented by a wide-ranging immunohistochemical panel and molecular analysis. Each of these methods requires extra tissue, underscoring the importance of obtaining an adequately sized sample for precise diagnosis [53]. A technique that offers insights into the disease type, subtype, and severity without requiring extra tissue sections could benefit from being considerably more tissue-conservative. MIR microscopic imaging is a promising diagnostic tool for lymphoma identification and subtyping. It could be particularly valuable in situations where there is an immediate clinical necessity for treatment or in instances of limited tissue samples.

The capability of MIR microscopy imaging to detect biochemical variations between reactive lymphadenopathy in VavBcl2/TACI-Ig mice and cancer in genetically altered Vav-Bcl2 mice was assessed in an animal model (Table 3). A strong correlation between MIR microscopy and multivariate image analyses was observed across mouse genotypes and morphological tissue characteristics obtained from hematoxylin and eosin (HE) staining. Furthermore, through PCA, spectral groupings from various phenotype samples can be differentiated, especially between follicular hyperplasia and cancer [54].

**Figure 3:**
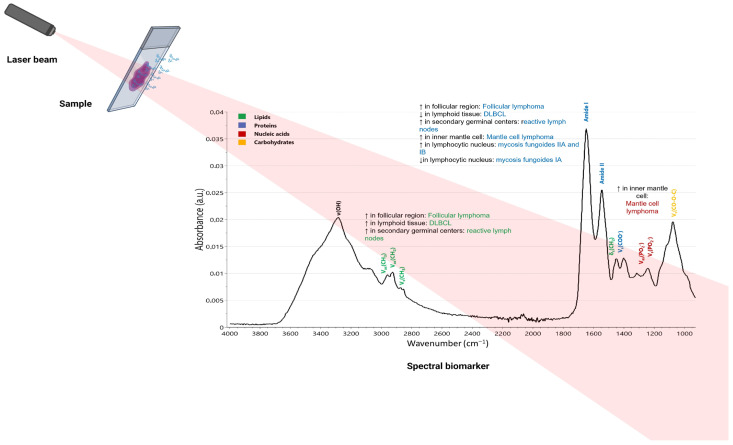
Infrared spectra illustrating differences among various hematological diseases. The spectra highlight distinct variations in protein signatures, evident from the amide I and II bands, as well as lipid content. The differences between the diseases are pronounced, emphasizing the potential of infrared spectroscopy as a diagnostic tool in hematological studies.

In a small study, including formalin-fixed and paraffin-embedded samples of six follicular lymphomas, twelve diffuse large B-cell lymphomas (DLBCLs), and reactive lymph nodes, MIR microscopic imaging could differentiate between the different entities. Additionally, it holds promise for subtyping lymphomas, as evidenced by the distinction between COO subtypes, proliferation levels, and programmed death-ligand 1 (PD-L1) expression. Follicular lymphomas exhibited an increased intensity of the amide I band and an increase in lipid-characteristic spectral features within the follicular region, but this was not seen in the interfollicular area. In contrast, DLBCL presented with reduced levels of amide I and increased, yet unevenly distributed, lipid amounts, which might indicate their distinct biological actions (Figure 3).

Reactive lymph nodes exhibited elevated levels of amide I and lipids within secondary germinal centers. In contrast, the adjacent areas showed reduced concentrations of both. The follicles manifested a certain polarity, notably characterized by a significantly lower presence of amide I and lipids on one side of the secondary follicle than on the other [55]. This aligns with the known histology and biology of secondary follicles [56]. Follicular lymphoma resembled normal germinal centers and, as a result, was rich in amide I and lipids within the follicular section. Through PCA, it was possible to differentiate various tissue types, not only distinguishing between reactive lymph nodes and lymphomas but also between indolent and aggressive lymphomas. PCA could also represent both COO subtypes of DLBCL and differentiate between nodal and extranodal diseases, although the cluster distinctions were not as clear-cut as those of the others. One potential benefit of MIR is its ability to obtain results in just a few hours, which is potentially quicker than immunohistochemistry, provided that the right technical equipment is on hand. Patients in dire and immediate need of correct treatment may find this method advantageous. Moreover, MIR imaging requires only a single, unstained slide. As a result, this technique might be especially valuable when there is limited tissue for diagnosis or subtyping and it could even be adapted for cytological samples [55]. Additionally, MIR imaging of unstained tissue slides, combined with a deep learning strategy utilizing a convolutional autoencoder alongside both supervised and unsupervised training techniques, can distinguish between benign and malignant lymphoid tissues, as well as categorize lymphoma subtypes. This could serve as an adjunct method for the rapid initial evaluation of the type and severity of the disease. It may aid in the early identification of pressing diagnoses and in prioritizing these patients in the standard workflow [57].

In a non-Hodgkin EL4 lymphoma mouse model, ATR-FTIR spectroscopy of dried serum samples revealed distinct differences in the spectra between control and tumorous samples. These differences, primarily in the absorption intensities of proteins, carbohydrates, and nucleic acids, mirror findings from serological tests that highlight changes in specific proteins, peptides, and nucleic acids in patients with lymphoma. These variations in biomolecules or serological markers are likely attributed to tumor-induced changes, helping to identify spectral markers [58]. Hands et al. first used ATR-FTIR spectroscopy to distinguish brain cancer from noncancer patients with high accuracy [59], whereas Cameron et al. differentiated between glioblastoma and primary cerebral lymphoma [60]. Analysis of IR spectra from 641 blood serum samples from brain cancer patients and healthy controls demonstrated that ATR-FTIR spectroscopy could effectively differentiate between both groups with over 90% accuracy. It identified nuanced differences in protein structures between patient groups using amide I deconvolution. Furthermore, various brain lesions, including glioblastoma, meningioma, primary central nervous system lymphoma, and metastasis, could be distinguished with accuracies exceeding 80% [61]. Subsequent studies showed that removing high-molecular-weight components from patient serum reduced the accuracy of cancer discrimination, emphasizing their importance in diagnostics and simplifying sample preparation [62].

When juxtaposed with visual assessment, which is the prevailing benchmark in routine diagnostics, visible and near-infrared (Vis-NIR) hyperspectral imaging is dependable for ascertaining the percentage of PD-L1^+^ cells in human lymphoma through brightfield-based immunohistochemistry. Hyperspectral imaging consistently detects PD-L1^+^ cells, which is comparable to digital image analysis. In contrast to traditional digital image analysis, hyperspectral imaging may present certain benefits, such as the capacity to analyze multiple stains of co-localized antigens or to pinpoint and measure disparities in staining intensities. Hyperspectral imaging is adept at distinguishing varying color tones, and its ability to capture multiple spectra can enhance the differentiation of overlapping staining patterns or diverse staining intensities in a bright field. Moreover, hyperspectral imaging provides an avenue for a more profound understanding of the biochemical makeup of tissues, yielding essential supplementary data [63]. Hyperspectral imaging can measure protein expression, such as the Ki67 proliferation index, as effectively as traditional digital imaging analysis. As hyperspectral imaging taps into the NIR spectrum to potentially provide more details on the biochemical makeup of pathological samples, it could be valuable for analyzing multiplex immunohistochemistry, especially when overlapping reactions occur [64].

Mantle cell lymphoma (MCL) is a mature B-cell lymphoma that arises in the inner mantle zone. This is marked by increased levels of cyclin D1 due to 11q13 translocation [65]. MCL exhibits diverse characteristics, with various morphological forms identified: the classic type as well as more aggressive variants, such as blastoid and pleomorphic mantle cell lymphomas [66,67]. The division of MCL into classic and aggressive subtypes through histopathological methods is firmly rooted in the biochemical profiles of both categories, as demonstrated by a combination of S-FTIR microscopy and PCA. In both malignant tissues, there was a notable increase in the absorbance for peaks linked to amide I, amide II, and nucleic acids. This was particularly prominent in patients with aggressive MCL. A wavenumber shift was detected for PO^2−^ asymmetric stretching vibrations associated with DNA. PCA loadings highlighted that amide I was rich in β-sheet structures, while amide II bands primarily distinguished the two MCL subtypes. These data align with existing information on protein overexpression in more aggressive MCL variations [68]. FTIR spectroscopy for single-cell analysis in MCL revealed notable insights into lipid dysregulation in highly resistant cells. This led to the identification of a change in the CD36 receptor that affects the lipid balance [69].

The primary diagnostic challenge for cutaneous T-cell lymphoma (CTCL) lies in differentiating between early-stage malignant lymphoma and benign reactive lymphocytic infiltration. The early detection of cutaneous lymphoma is vital in clinical settings. In the early phases, distinguishing mycosis fungoides from other benign skin lymphoid infiltrations based solely on morphology might be unfeasible. Since the main malignant transformations in mycosis fungoides take place in the lymphocyte nucleus, biochemical shifts were investigated using S-FTIR microscopy with a high signal-to-noise ratio at a spatial resolution restricted by the diffraction (3–10 mm). Amide I/RNA and amide II/RNA ratios were higher in mycosis fungoides IIA and IB than in mycosis fungoides IA and pityriasis lichenoides chronica, with the distinctions among the three groups being notably significant [70]. This aligns with the findings of Andrus [71], which indicated that in vitro lymphoma cells exhibit increased RNA, protein, protein/RNA ratios, and lipid absorbance levels relative to regular lymphocytes, as deduced from the IR spectral variances between proliferating and nonproliferating lymphocytes.

**Table 3:** Spectral findings and wavenumber shifts in infrared studies of lymphoid malignancies.

Study Population	Main Technique	Key Spectral Findings	Ref
Animal model			
Transgenic mice model (VavBcl2/TACI-Ig mice, genetically altered Vav-Bcl2 mice)	MIR microscopy imaging	- Strong correlation between MIR microscopy and tissue characteristics. - Spectral groupings differentiate phenotypes (especially follicular hyperplasia and cancer). - Notable wavenumber shifts observed in amide I (1650 cm^−1^) and nucleic-acid-related bands.	[54]
EL4 mouse model	ATR-FTIR spectroscopy	- Spectral differences between control and tumorous samples. - Variations in protein absorption intensities, with specific wavenumber shifts in the amide I band (1650 cm^−1^). - Changes in carbohydrate and nucleic acid bands.	[58]
**Human model**			
Follicular lymphomas, diffuse large B-cell lymphomas, reactive lymph nodes	MIR imaging	- Differentiation between lymphoma entities. - Subtyping based on wavenumber shifts. - Follicular lymphoma: higher concentration of amide I proteins and lipids in follicular region (1650 cm^−1^), but not in the interfollicular area. -Diffuse large B-cell lymphomas: lower amounts of amide I and a higher but more heterogeneously distributed amounts of lipids. - Reactive lymph nodes: high amounts of amide I and lipids in the secondary germinal centers, with lower amounts in the surrounding areas. - The follicles exhibit a distinct polarity, particularly evident in the asymmetric distribution of biochemical components. One side of the secondary follicle displays a significantly lower intensity of the amide I band and lipid-related spectral features compared to the opposite side. This asymmetry aligns with the histological and biological characteristics of secondary follicles.	[55]
Human lymphoid tissues, benign and malignant lymphoid tissues	MIR imaging	- Distinguishes benign and malignant lymphoid tissues. - Categorizes lymphoma subtypes based on specific wavenumber shifts.	[57]
Human lymphoma (percentage of PD-L1^+^ cells)	Visible and NIR hyperspectral imaging	- Differentiates lymphoma subtypes based on spectral signatures. - Measures protein expression with specific wavenumber shifts around 1650 cm^−1^. - Potential for multiplex immunohistochemistry analysis.	[63]
Mantle cell lymphoma	S-FTIR microscopy	- Increased absorbance for peaks linked to amide I (1650 cm^−1^), amide II, and nucleic acids. - Notable wavenumber shifts in DNA vibrations. - Distinguishes classic and aggressive MCL subtypes.	[68]
Cutaneous T-cell lymphoma	S-FTIR microscopy	- Higher amide I/RNA and amide II/RNA ratios in mycosis fungoides IIA and IB compared to MF IA and pityriasis lichenoides chronica (around 1650 cm^−1^). - Distinctions among the three groups based on specific wavenumber shifts.	[70]

Abbreviations: MCL, mantle cell lymphoma; MF, mycosis fungoides; NIR, near-infrared; S-FTIR microscopy, synchrotron radiation Fourier transform infrared microscopy.

### 3.4. Thalassemia

Thalassemia is a genetic disorder related to Hb production that affects individuals from various regions worldwide. Hematological phenotypic analysis serves as an initial screening technique for diagnosing thalassemia. This complexity arises from the need for various chemical agents and diverse measurement instruments. Quick screening and subsequent categorization of thalassemia have significant clinical importance. NIR spectroscopy has been utilized for the swift assessment of preliminary thalassemia screening markers (mean corpuscular hemoglobin (MCH), mean corpuscular volume (MCV)) related to Hb using moving-window partial least squares (MW-PLS) for wavelength selection (Table 4) [72]. Chen et al. advanced the wavelength selection technique to equidistant combination PLS (EC-PLS) and further crafted a swift analysis of HbA2 for β-thalassemia screening using NIR spectroscopy. The relative content marker HbA2 was indirectly measured by concurrently analyzing two absolute content markers (Hb and Hb∙HbA2). During the validation process, the predicted values for the relative percentage of HbA2 were derived from the anticipated Hb and HbA2 values. Both the sensitivity and specificity for β-thalassemia were 100%, suggesting that HbA2 prediction is extremely precise for identifying β-thalassemia. The suggested NIR approach is quick, straightforward, and promising for mass thalassemia screening. By employing quantitative molecular spectroscopic evaluations of two absolute content markers to indirectly measure a relative content indicator, this strategy could potentially be adapted for other applications [73].

Changes in the microenvironment of the thalassemia bone marrow during ineffective erythropoiesis can lead to variations in bone marrow mesenchymal stem cells, which has been examined by ATR-FTIR spectroscopy. There was a noticeable increase in the content of lipids, proteins, glycogen, and nucleic acids in thalassemic bone marrow mesenchymal stem cells compared to healthy bone marrow mesenchymal stem cells. This has been linked to increased cell growth and bone marrow activity during ineffective erythropoiesis. In contrast, post-transplant bone marrow mesenchymal stem cells showed a marked reduction in these macromolecules when compared to pre-transplant bone marrow mesenchymal stem cells, suggesting that bone marrow transplantation therapy aids in rectifying the effects of ineffective erythropoiesis and compromised bone marrow environment. These changes were further supported by enzyme-linked immunosorbent assay (ELISA) results for erythropoietin and growth differentiation factor 15 in bone marrow plasma samples, which reflect the state of ineffective erythropoiesis, and by the MTT proliferation test on bone marrow mesenchymal stem cells. These variations allowed for distinct categorization of the sample groups through cluster analysis. These findings offer insights for research focusing on the interplay between hematopoietic and mesenchymal stem cells in the bone marrow [74]. In addition, ATR-FTIR spectroscopy has been used in quantitative studies of Hb, MCH, and MCV [75,76]. The optimal wavebands have been determined using the enhanced MW-PLS method, considering stability and consistency across various calibration and prediction sets. The best MW-PLS wavebands identified were 1722–1504 cm^−1^ for Hb, 1653–901 cm^−1^ for MCH, and 1562–964 cm^−1^ for MCV. A model closely matching the ideal MW-PLS is suggested for each parameter. The waveband for a similar model of Hb was 1717–1510 cm^−1^, while MCH and MCV shared a waveband of 1562–901 cm^−1^. All chosen wavebands fell within the MIR fingerprint region, delivering excellent validation results in their respective models. With the optimal wavebands, the sensitivity was 100% and specificity was 96.9%, while with the alternative bands, they were 100% and 95.3%, respectively. Hence, this spectral assessment offered precise results in thalassemia screening, outperforming traditional methods in terms of speed and simplicity. It has great potential for mass thalassemia screening [75]. The IR spectra highlighted differences in the secondary structure of Hb between patients with β-thalassemia and controls. Specifically, there was a reduction in the α-helix content (1657 cm^−1^), an increase in both parallel and antiparallel β-sheets (1640 and 1680 cm^−1^, respectively), and alterations in the absorption band of the tyrosine ring (1517 cm^−1^). Furthermore, Hb from β-thalassemia patients exhibited a heightened intensity in the IR bands associated with the cysteine SH groups (2550 cm^−1^). Using unsupervised cluster analysis, which categorizes spectra based on minor IR spectral variances, it was possible to differentiate the Hb spectra of controls from those with β-thalassemia, largely due to differences in protein secondary structures (amide I and amide II bands (1400–1800 cm^−1^)). The total accuracy of the spectral classification exercise was 100% for the training set and 98% for the test set [77].

### 3.5. Sickle Cell Anemia

Sickle cell anemia is a hereditary condition that affects red blood cells, leading to persistent hemolytic anemia, diminished oxygen-carrying capabilities, compromised tissue oxygenation, and both acute and chronic organ damage, ultimately affecting the physical well-being of an individual.

NIR spectroscopy is a well-established, noninvasive technique for assessing tissue oxygenation (StO_2_). The microvascular Hb oxygen saturation, also known as tissue oxygen index (TOI), is diminished in individuals with sickle cell anemia (SS genotype). In the case of sickle cell HbC disease (SC genotype), this reduction is observed to a lesser degree [78,79,80,81,82,83]. In children with sickle cell disease, a reduction in both cerebral and muscle microvascular oxygenation was observed, mirroring the findings seen in adult patients with sickle cell disease (Table 5). Anemia, red blood cell rheology, and peripheral Hb oxygenation may collectively influence cerebral microvascular oxygenation in children with the SS genotype [80,84]. The cerebral TOI level in children with the SS genotype has also been linked to hematocrit levels, suggesting that the extent of anemia could influence microcirculatory oxygenation in the brain [85]. Although there was a positive correlation between cerebral TOI and RBC deformability in children with the SS genotype [86], similar to findings in adults [80], no impact of RBC deformability on muscle TOI was observed [86]. This finding aligns with prior observations in adults [67]. Significantly higher absolute cerebral flow and vasomotion were observed in the SS genotype group than in the AA and SC genotypes. This elevated cerebral vasomotion activity in individuals with SS may serve as an adaptive response by the cerebrovascular system to mitigate the effects of chronic cerebral hypoxemia, potentially safeguarding endothelial function [85]. Individuals with the SC genotype face a reduced risk of developing cerebral vasculopathy [87], a phenomenon that could be attributed to lower levels of hemolysis, milder anemia, and higher brain oxygenation. The elevated muscle TOI observed in children with the SC genotype, in contrast to those with the SS genotype, may contribute to the typically better physical exercise capabilities reported in the former group [88]. Although there were no discernible disparities in muscle vasomotion and flow motion among the three groups, heightened activity levels at the cerebral level in children with the SS genotype were detected when compared to both AA and SC genotypes. This finding aligns with that of a previous study conducted in adults with the SS genotype [80]. Enhanced vasomotion might serve as a compensatory mechanism to counteract the decreased cerebral TOI in individuals with the SS genotype [89,90].

In a small study involving 17 children and young adults diagnosed with sickle cell anemia and 13 control subjects, patients consistently exhibited lower cerebral StO_2_ levels throughout all stages of exercise and experienced significantly more significant declines in cerebral StO_2_ as exercise progressed. When comparing patients and controls at equivalent workloads, patients displayed lower cerebral StO_2_ levels (69.2 ± 6.6% vs. 79.5 ± 5.3%, *p* < 0.001) [85]. The decrease in cerebral StO_2_ levels during exercise could have more significant implications for individuals with sickle cell anemia, as hypoxia-induced sickling is known to play a role in cerebral vasculopathy and the risk of stroke [91]. Although reduced cerebral StO_2_ has been associated with neurocognitive impairment [92], research in children is scarce, and a defined minimum threshold for clinical relevance has yet to be established. Changes in cerebral StO_2_ during exercise in sickle cell anemia patients may be elucidated by the physiology of cerebral hemodynamics [93]. In sickle cell anemia, individuals respond to diminished arterial oxygen levels by augmenting cerebral blood flow and oxygen extraction [93,94]. However, as compensatory mechanisms become depleted, cerebral tissue oxygenation can be affected [91]. The substantial decline in cerebral StO_2_ during the later phases of exercise in sickle cell anemia patients suggests the possibility of compensatory mechanisms becoming overwhelmed or insufficient [85]. In contrast to previous findings, an initial rise in cerebral StO_2_ was observed, which was subsequently followed by a decrease back toward the baseline level as exercise intensity reached its highest point [95]. Discrepancies in outcomes may arise from variations in exercise testing procedures or individual patient-related factors such as baseline fitness, training history, and underlying medical conditions. Quadriceps StO_2_ levels increased similarly in both patients and controls during the early stages of exercise. However, patients demonstrated more pronounced decreases from their baseline values as exercise continued. When assessing patients and controls at matched workloads, patients exhibited a trend toward reduced quadriceps StO_2_ levels (67.7 ± 9.0% vs. 73.2 ± 7.9%, *p* = 0.09) [85]. Sickle cell anemia is linked to changes in skeletal muscles, such as reduced capillary density and muscle atrophy in its initial state [96]. Research focused on the skeletal muscle of individuals with sickle cell anemia could offer further understanding of the potential reasons for the diminished StO_2_ levels observed during exercise [85]. In a comparative study involving children and adolescents, microvascular oxygenation in both cerebral and muscle tissues was significantly lower in individuals with the SS genotype than in those with the SC genotype. Furthermore, among children with the SS genotype, a correlation was found between cerebral TOI and several key factors including hematocrit levels, red blood cell deformability, and SpO_2_. Additionally, absolute cerebral vasomotion and flow motion were markedly increased in individuals with the SS genotype compared to both AA and SC children. Interestingly, these differences were not observed at the muscle tissue level [86].

### 3.6. Myelodysplastic Syndrome

Myelodysplastic syndrome (MDS) is a condition stemming from abnormal hematopoietic stem cells, marked by deficiencies in peripheral blood due to unsuccessful blood cell production, potentially resulting in bone marrow failure [97]. For the diagnosis of MDS, prolonged cytopenia must be present without any rectifiable causes, such as nutritional deficiencies. Diagnosis can be confirmed if any of the following conditions are met: (1) the existence of single- or multilineage cytologic dysplasia in at least 10% of cells, (2) evidence of elevated myeloblasts ranging from 5% to 19%, or (3) a particular cytogenetic anomaly when there is ongoing unexplained cytopenia [98,99,100,101].

Using FTIR statistical models, MDS granulocyte DNA was differentiated from normal granulocyte DNA. This ability stems from the observable structural variances in the average DNA spectra between the MDS and control groups. The presence of these structural variations enabled the creation of a discriminant analysis model that was forecast with high sensitivity (100%) and specificity (95%) using a cutoff point of ≥0.4. When comparing the average DNA spectra of the control group (n = 20) to the MDS samples (n = 10), notable statistical differences were found (*p* < 0.05) across 43.4% of the overall spectral range (1750–700 cm^−1^) [102]. The observed disparities are indicative of the in-plane ring and C=N stretching oscillations of cytosine, observed at 1651 cm^−1^ [103]. This includes the antisymmetric stretching motions of the PO^2−^ structure at 1230 cm^−1^, the symmetric stretching oscillations of the PO^2−^ structure at 1084 cm^−1^, and the C-O stretching motions attributed to ribose/deoxyribose at 1062 cm^−1^. Beyond the primary differences, a noteworthy disparity was identified at the subtle peak of 1481 cm^−1^. This corresponds to the in-plane oscillations of base residues in the context of the N-H and C-H deformation patterns. Discrepancies in multiple subtle peaks were also detected within the 950–760 cm^−1^ range, representing assorted ribose-phosphate core chain oscillations. The notable spectral variations identified across a considerable segment of the spectral range unequivocally show that the change from control granulocyte DNA to the MDS structure encompasses clear changes in both the nucleotide bases and backbone [102].

### 3.7. Myeloproliferative Neoplasms

#### 3.7.1. Primary Myelofibrosis

Primary myelofibrosis is categorized as a myeloproliferative neoplasm distinguished by the presence of clonal neoplasms originating from stem cells. It is a hematological disorder characterized by damage to the blood cells, bones, lymph nodes, spleen, and liver. Neoplasms are the predominant cause of the disease, primarily affecting blood tissues [104]. The diagnosis of primary myelofibrosis relies on various factors, including physical examination, peripheral blood observations, bone marrow morphology, cytogenetic assessments, and the identification of molecular markers. In this context, gene mutations are frequently used.

Analysis using FTIR spectroscopy on dried blood serum revealed that individuals with primary myelofibrosis exhibit elevated levels of phospholipids and proteins, along with a reduction in H-O=H vibrations, which was visually evident (Table 6). PCA demonstrated the potential to distinguish between dried blood serum samples obtained from patients with primary myelofibrosis and those from healthy individuals. Utilizing the grid search support vector machine (GS-SVM) model, the prediction accuracy ranged from 0.923 to 1.00, depending on the FTIR range under examination. Moreover, it was observed that the ratio of α-helix to β-sheet structures in proteins was 1.5 times higher in patients with primary myelofibrosis than in healthy controls. The vibrations associated with the C–O bond and amide III region of proteins exhibited the highest probability values, signifying significant alterations in these spectral features among primary myelofibrosis patients compared to those with normal spectra [105].

#### 3.7.2. Essential Thrombocythemia

Essential thrombocythemia arises from the transformation of versatile hematopoietic stem cells, although its molecular underpinnings are not yet fully understood. However, tyrosine kinases, particularly Janus kinase 2 (JAK2), have been linked to myeloproliferative diseases other than chronic myeloid leukemia [113]. Essential thrombocythemia is a distinctively varied rare tumor that can evolve into myelofibrosis or leukemia. Its manifestations can range from silent thrombocytosis to heightened thrombotic or hemorrhagic conditions [114,115]. Currently, diagnosis relies on bone marrow tests, *JAK2* mutation studies, and various clinical indicators; however, each has its own constraints [116].

Using FTIR-spectra-based machine learning techniques and chemometrics, FTIR spectroscopy analysis was conducted on the blood serum of 86 patients and 45 healthy volunteers who served as controls. In patients with essential thrombocythemia, there was a decreased protein level paired with an increased lipid level compared to the controls. The support vector machine discriminant analysis (SVM-DA) model demonstrated 100% accuracy in calibration sets for both spectral regions and accuracies of 100% and 96.4% for the prediction sets in the 800–1800 cm^−1^ and 2700–3000 cm^−1^ spectral regions, respectively. Dynamic spectrum alterations revealed that CH_2_ bending and amide II and C-O vibrations could act as spectroscopic markers for essential thrombocythemia. Chemical variations in the serum obtained from patients with essential thrombocytopenia revealed an elevated presence of amide I and amide III vibrations, coupled with a reduced level of amide II. This may indicate alterations in the protein fraction, possibly resulting from mutations. Considering that both PCA and the variations in the absorption dynamics of the FTIR spectrum indicate that amide II is the most likely wavenumber to serve as an essential thrombocytopenia marker, these specific functional groups could be employed in spectroscopic diagnosis. Finally, a positive correlation was observed between FTIR peaks and the initial degree of bone marrow fibrosis, along with the absence of the *JAK2 V617F* mutation [106]. These peaks predominantly arose from the stretching vibrations of the phosphate groups of DNA and RNA (1079 cm^−1^) and the bending motions of the peptide backbone (1241 cm^−1^). They also corresponded to the C-H bending of CH_3_ groups found in proteins, lipids, and carbohydrates (1307 cm^−1^), CH_2_ bending in proteins and lipids (1453 cm^−1^), and C=O stretching in amide II (1537 cm^−1^) and amide I (1637 cm^−1^). Moreover, asymmetric (2865 cm^−1^) and symmetric (2928 cm^−1^) stretching of CH_2_ groups across proteins, lipids, and carbohydrates were observed. Finally, the 2964 cm^−1^ peak is linked to the C-H stretching of CH_3_ groups in proteins, lipids, and carbohydrates [107,108,109]. This implies that alterations in the concentrations or makeup of specific biomolecules, such as DNA, RNA, proteins, lipids, and carbohydrates, may correlate with the onset of essential thrombocythemia and progression of essential thrombocythemia. Some FTIR peaks might be linked to the early stages of the disease, while the existence of the *JAK2 V617F* mutation and certain other FTIR peaks might be linked to a more progressive phase of the disease. These FTIR peaks have the potential to act as biomarkers for essential thrombocytopenia. Additional research is essential to confirm these observations and assess the clinical relevance of FTIR spectroscopy in diagnosing and treating essential thrombocytopenia [106].

#### 3.7.3. Chronic Myeloid Leukemia

Chronic myeloid leukemia (CML) is a type of cancer that starts in the blood-forming cells of the bone marrow and invades the blood. Its progression and treatment have been the focus of extensive research over the years. A critical aspect of treating CML is understanding the molecular changes and resistance mechanisms that these cancerous cells develop against therapies. In the context of CML, FTIR has been employed to probe the changes related to drug resistance, offering a deeper understanding of the progression of the disease and the challenges associated with its treatment. FTIR also offers an appropriate approach for examining structural alterations related to drug resistance in leukemia. Imatinib is a potent tyrosine kinase inhibitor that has shown favorable outcomes as a primary treatment for patients with CML. However, there is a challenge where CML cells can bypass the apoptotic effects of imatinib and develop resistance, presenting a significant clinical hurdle to achieving successful outcomes.

Alterations in macromolecules between the original and imatinib-resistant K562/IMA-3 cells have been analyzed using FTIR spectroscopy. Resistance to imatinib led to observable changes, including a reduction in glycogen levels (a decrease in intensity at 1155 cm^−1^) and heightened membrane order (2959 cm^−1^) [110]. Cancer cells are known for their heightened carbohydrate consumption due to their increased energy needs, a phenomenon termed the Warburg effect. The diminished intensity of these two bands may be attributed to the decreased presence of polysaccharides in resistant cells [111]. Likewise, it is theorized that drug resistance demands more energy owing to the added responsibility of removing toxic substances and medications. There was a noticeable increase in unsaturated lipids (wavenumber 3015 cm^−1^) in imatinib-resistant cells, indicating lipid peroxidation. Drug-resistant cells appear to have heightened resistance to reactive oxygen species (ROS), such as peroxides. This resistance also results in a shift in the lipid-to-protein ratio, with the protein content increasing relative to that of nucleic acids, suggesting enhanced transcription and protein production. Frequency alterations in nucleic acid bands (wavenumbers 1239 cm^−1^, 1086 cm^−1^, and 971 cm^−1^) highlighted structural and organizational modifications in the cell nucleus. The variations in the band intensities at 1239 and 970 cm^−1^ for the imatinib-resistant cells suggested alterations in the nuclear structure and arrangement. These include changes in the nucleus-to-cytoplasm ratio, chromatin clustering, and differences in DNA condensation between imatinib-sensitive and imatinib-resistant K562 cells. Distinct variations in the amide bands (wavenumbers 3300 cm^−1^ (amide I) and 3061 cm^−1^ (amide II)) indicated proteomic alterations in resistant cells. Furthermore, while the structure of antiparallel beta-sheets grew (wavenumber 1690 cm^−1^), the structures of the alpha-helix (wavenumber 1653 cm^−1^), beta-sheets (wavenumber 1637 cm^−1^), random coils (wavenumber 1648 cm^−1^), and turns declined in these cells [110].

#### 3.7.4. Polycythemia Vera

Polycythemia vera is a Philadelphia-chromosome-negative myeloproliferative disorder that leads to enhanced bone marrow activity. This challenging condition is marked by excess production of red blood cells, although it can also cause an increase in white blood cells and platelets [117]. Micro-FTIR spectroscopy has been used to study the behavior and treatment efficacy of patients with polycythemia vera undergoing alpha2a-interferon (α2a-IFN) therapy. A spectroscopic metric (A_1_/A_2_) was employed, representing the proportion between the integrated areas of the bands at 1080 cm^−1^ and 1540 cm^−1^, which are attributed to nucleic acids and protein components, respectively. This was determined based on the spectra of the individual megakaryocytes [112].

## 4. Future Directions and Potential Developments

In the rapidly evolving world of medical diagnostics, IR spectroscopy could be a game-changer for hematological disease detection. As we stand at the threshold of this transformative era, various prospective developments and anticipated advancements loom on the horizon. The next generation of IR spectroscopy devices, driven by cutting-edge technological innovations, are expected to be characterized by heightened sensitivity, unparalleled precision, and swift operational speeds. One of the critical anticipated advancements is the achievement of a superior spatial resolution. This feature, which allows for the differentiation of even minute spectral variations, will be instrumental in unmasking intricate cellular heterogeneities, thereby offering researchers and clinicians an unparalleled depth in their investigations. Furthermore, the potential synergy between IR spectroscopy and computational methodologies is exciting. As artificial intelligence and machine learning continue to mature, their integration with spectroscopic techniques may set the stage for a new era of diagnostic precision. These computational tools, capable of processing and analyzing vast spectral datasets, hold the promise of not only enhancing diagnostic accuracy but also providing prognostic insights and predicting disease trajectories.

The democratization of IR technology is another promising prospect. The future has seen the emergence of compact, portable, and user-friendly IR devices. Such innovations would make this advanced diagnostic tool accessible beyond the confines of specialized labs, integrate it seamlessly into point-of-care settings, and make real-time diagnostics a tangible reality. In addition, as the scientific community continues to unravel the complexities of hematological pathologies, there is a compelling need for interdisciplinary collaboration. The synergy among molecular biologists, seasoned clinicians, and expert spectroscopists could result in groundbreaking discoveries. These joint ventures could potentially spearhead tailored therapeutic interventions, wherein spectral data are the cornerstone of personalized patient-care strategies.

However, with great potential comes the inherent responsibility of ensuring that technology is used judiciously. As the integration of IR spectroscopy into hematological diagnostics becomes more profound, the scientific community will be tasked with the critical mandate of establishing robust standardized protocols, rigorous calibration methods, and comprehensive data interpretation guidelines. These measures are essential in ensuring that spectral insights can be replicated, validated, and meaningfully translated into actionable clinical outcomes.

To summarize, the progression of IR spectroscopy in the domain of hematological diagnostics, although already marked by significant milestones, still has a long and promising path ahead. The canvas is vast, and the opportunities are myriad. If navigated with vision and diligence, this era has the potential to redefine hematological patient care in ways hitherto unimagined. However, to date, research on IR spectroscopy in hematological disorders has been limited to a small number of studies, often involving a modest number of patients. Thus, larger studies are needed to confirm these initial findings and to further explore the potential of this technology in this domain.

## 5. Conclusions

IR spectroscopy, once primarily associated with chemistry and physics, has evolved into a significant tool in the field of medical diagnostics. Its ability to offer noninvasive and swift molecular insights has made it a groundbreaking tool in contemporary medicine. The distinctive “spectral fingerprints” produced by this technique now reveal the subtle complexities of various hematological conditions (Table 7), providing clinicians with a profound depth for diagnostic analysis. Although the potential of IR spectroscopy has been well established, we are still in the early stages of unlocking its full capabilities. Technological enhancements combined with collaborations across various scientific disciplines are expected to further elevate their influence. However, like many innovations on the cusp of broader adoption, there are challenges to surmount. Standardization, nuances of data interpretation, and seamless integration into comprehensive medical systems will be paramount for achieving enduring success. Given its current trajectory and clear advantages, there is a strong belief that IR spectroscopy is on track to shape the future of hematological disease diagnosis significantly, ushering in an era of enhanced precision, tailored treatments, and improved patient outcomes. Given that the IR spectra of tissues can be employed to teach techniques to identify their biochemical signatures, IR imaging and spectroscopy may assist pathologists in instances where discerning closely related cell states is challenging.

## Figures and Tables

**Table 2:** Infrared spectroscopy studies revealing key spectral findings in chronic lymphocytic leukemia.

Study Population	Sample and Main Technique	Key Spectral Findings	Ref
CLL patients	CLL cells vs. normal cells Micro-FTIR spectroscopy	- Main spectral shifts in leukemic cells in the range between 1000 and 1200 cm^−1^, correlated with nucleic acids (DNA/RNA). - Consistent correlation between the decline in nucleic acid content and the decrease in blast percentage post-chemotherapy in both B- and T-cell patients.	[27]
CLL patients	CLL cells vs. normal cells FTIR spectroscopy	- Higher DNA content (observed in spectral range 900–1300 cm^−1^) and lower lipid content in CLL cells. - Segregation of CLL cells into three distinct subgroups based on spectral variations. - Increased DNA content associated with chromosomal abnormalities.	[24]
CLL patients vs. healthy individuals	Human plasma Micro-FTIR spectroscopy	- Spectral peaks at 1056 cm^−1^ (carbohydrates), 1270 cm^−1^ (amide III band), and 1592 cm^−1^ (δ(NH_2_): amino acids) exhibited significant reductions in patient samples. - Effective classification of healthy and patient samples based on these spectral changes.	[49]

Abbreviations: CLL, chronic lymphocytic leukemia; micro-FTIR spectroscopy, Fourier transform infrared microspectroscopy.

**Table 4:** Infrared spectroscopy insights into beta-thalassemia.

Study Population	Main Technique	Key Spectral Findings	Ref
Patients with beta-thalassemia major	ATR-FTIR spectroscopy and ELISA	- Increased content of macromolecules. - Marked reduction post-transplant. - Varied erythropoietin and GDF-15 levels.	[74]
Individuals screened for thalassemia	ATR-FTIR spectroscopy	- Optimal wavebands: 1722–1504 cm^−1^ for Hb, 1653–901 cm^−1^ for MCH, and 1562–964 cm^−1^ for MCV. - Alternative bands: 1717–1510 cm^−1^ for Hb, and 1562–901 cm^−1^ for MCH and MCV.	[75,76]
β-thalassemia patients vs. controls	ATR-FTIR spectroscopy	- Reduction in α-helix content (1657 cm^−1^), increase in β-sheets (1640 cm^−1^ and 1680 cm^−1^), alterations in tyrosine ring absorption (1517 cm^−1^), and heightened intensity in bands associated with cysteine SH groups (2550 cm^−1^).	[77]

Abbreviations: ATR-FTIR spectroscopy, attenuated total reflectance–Fourier transform infrared spectroscopy; ELISA, enzyme-linked immunosorbent assay; GDF-15, growth differentiation factor 15; MCH, mean corpuscular hemoglobin; MCV, mean corpuscular volume.

**Table 5:** NIR-spectroscopy-derived metrics of cerebral and muscular oxygenation in sickle cell disease.

Study Population	Main Technique	Key Spectral Findings	Ref
Individuals with sickle cell anemia and sickle cell hemoglobin C disease	NIR spectroscopy	- Reduction in TOI and cerebral/muscle microvascular oxygenation. - Elevated cerebral vasomotion activity in SS individuals as a possible adaptive response to chronic cerebral hypoxemia.	[78,79,80,81,82,83,84,85,86,87,88]
Children and young adults with sickle cell anemia vs. control subjects	NIR spectroscopy	- Lower cerebral StO_2_ levels in patients during all exercise stages. - More pronounced declines in cerebral StO_2_ as exercise progressed. - A trend toward reduced quadriceps StO_2_ levels in patients.	[85,91,92,93,94,95]
Comparative study involving children and adolescents with SS and SC genotypes	NIR spectroscopy	- Significantly lower microvascular oxygenation in cerebral and muscle tissues in SS individuals. - Correlation between cerebral TOI, hematocrit levels, red blood cell deformability, and SpO_2_ in SS individuals.	[86]

Abbreviations: NIR, near-infrared; SC, sickle cell hemoglobin C; SpO_2_, saturation of peripheral oxygen; SS, sickle cell; StO_2_, oxygen saturation; TOI, tissue oxygenation index.

**Table 6:** Fourier transform infrared spectroscopy in myeloproliferative disorders: a detailed examination of spectral markers and molecular variations.

Study Population	Main Technique	Key Spectral Findings	Ref
Individuals with primary myelofibrosis	FTIR spectroscopy	- Elevated levels of phospholipids and proteins; reduction in H-O=H vibrations; ratio of α-helix to β-sheet structures in proteins is 1.5 times higher; significant alterations in vibrations associated with the C–O bond and the amide III region of proteins.	[105]
Patients with essential thrombocythemia vs. control subjects	FTIR spectroscopy with machine learning techniques	- Decreased protein and increased lipid levels. - Spectroscopic markers: CH_2_ bending, amide II, and C-O vibrations; elevated presence of amide I and amide III vibrations, reduced level of amide II; FTIR peaks at 1079 cm^−1^, 1241 cm^−1^, 1307 cm^−1^, 1453 cm^−1^, 1537 cm^−1^, 1637 cm^−1^, 2865 cm^−1^, 2928 cm^−1^, and 2964 cm^−1^ representing vibrations from DNA, RNA, proteins, lipids, and carbohydrates.	[106,107,108,109]
Original and imatinib-resistant K562/IMA-3 cells (chronic myeloid leukemia)	FTIR spectroscopy	- Reduction in glycogen levels (1155 cm^−1^); heightened membrane order (2959 cm^−1^); increase in unsaturated lipids (3015 cm^−1^); frequency alterations in nucleic acid bands (1239 cm^−1^, 1086 cm^−1^, 971 cm^−1^); proteomic alterations in resistant cells with variations in amide bands (3300 cm^−1^ for amide I and 3061 cm^−1^ for amide II); structure changes in antiparallel beta-sheets (1690 cm^−1^), alpha-helix (1653 cm^−1^), beta-sheets (1637 cm^−1^), random coils (1648 cm^−1^), and turns.	[110,111]
Polycythemia vera patients undergoing α2a-IFN therapy	Micro-FTIR	Spectroscopic metric (A₁/A₂) based on integrated areas of bands at 1080 cm^−1^ (nucleic acids) and 1540 cm^−1^ (protein components).	[112]

Abbreviations: IFN, interferon; micro-FTIR spectroscopy, Fourier transform infrared microspectroscopy.

**Table 7:** Summary of infrared spectroscopic variations in hematological diseases.

Hematological Disease	Spectral Fingerprint Features	Explanation of Features	Ref
Leukemia	- DNA marker bands at 966 cm^−1^ and 530 cm^−1^. - H2959 cm^−1^/H2931 cm^−1^ ratio. - RNA/DNA ratios at 1115 cm^−1^/1028 cm^−1^.	- Characteristic of lymphoid leukemia. - Indicates significant differences between leukemia patients and healthy individuals.	[23]
ALL	- Reduction in protein content (amide II band changes). - Variations in nucleic acids (1000–1200 cm^−1^ region). - Lipid and protein changes (2800–3000 cm^−1^ region).	- Signifies alteration in lymphocyte composition. - Reflects biochemical alterations from chemotherapy and disease progression.	[27]
AML	- Changes in protein structures (α-helices at 1657 cm^−1^ and 1650 cm^−1^; β-sheets at 1686 cm^−1^ and 1635 cm^−1^). - Amino acid alterations.	- Indicates a reduction in α-helical protein structures and an increase in β-sheets. - Reflects specific biochemical changes associated with AML.	[41]
CLL	- Higher DNA content and lower lipid content. - Spectral peaks at 1056 cm^−1^, 1270 cm^−1^, and 1592 cm^−1^.	- Highlights differences in cellular composition of CLL cells compared to normal cells.	[49]
CML	- Reduction in glycogen levels (1155 cm^−1^). - Heightened membrane order (2959 cm^−1^). - Increase in unsaturated lipids (3015 cm^−1^). - Proteomic alterations (3300 cm^−1^ for amide I, 3061 cm^−1^ for amide II).	- Indicates metabolic and structural changes in imatinib-resistant cells. - Suggests alterations in lipid composition and protein structure.	[110,111]
Lymphoma	- Variations in amide I band and lipid distribution. - Specific changes in protein secondary structure (β-sheet protein constituents at 1688 cm^−1^).	- Differentiates between follicular lymphomas and DLBCL. - Indicates changes in cellular protein composition.	[36]
Thalassemia	- Increase in macromolecules in bone marrow mesenchymal stem cells. - Changes in Hb secondary structure (α-helix reduction at 1657 cm^−1^, β-sheets increase at 1640 and 1680 cm^−1^).	- Reflects increased cell growth and bone marrow activity. - Indicates alterations in hemoglobin structure.	[77]
MDS	- Structural variances in DNA spectra. - Peaks at 1651 cm^−1^, 1230 cm^−1^, and 1084 cm^−1^.	- Indicates changes in nucleotide bases and backbone, differentiating MDS from normal cells.	[103]
Essential thrombocythemia	- Decreased protein and increased lipid levels. - Amide I (1637 cm^−1^) and amide III vibrations. - Reduced level of amide II (1537 cm^−1^). - Peaks related to DNA and RNA (1079 cm^−1^), peptide backbone (1241 cm^−1^), CH_3_ groups (1307 cm^−1^), CH_2_ bending (1453 cm^−1^), and C-H stretching (2865 cm^−1^, 2928 cm^−1^, 2964 cm^−1^).	- Reflects biochemical changes in the serum of patients. - Indicates alterations in protein and lipid composition, possibly resulting from mutations.	[106,107,108,109]

Abbreviations: ALL, acute lymphatic leukemia; AML, acute myeloid leukemia; CLL, chronic lymphatic leukemia; CML, chronic myeloid leukemia; DLBCL, diffuse large B-cell lymphoma; MDS, myelodysplastic syndrome.

## Data Availability

Not applicable.
